# A skills-first revival for professional public health education

**DOI:** 10.1093/ije/dyag138

**Published:** 2026-08-04

**Authors:** Charles C Branas

**Affiliations:** Gelman Professor and Department Chair of Epidemiology, Columbia University Mailman School of Public Health, New York, United States

Recently, I met with an undergraduate public health major at a liberal arts college who was weighing up whether to apply to master’s in public health (MPH) programs for training in epidemiology. Their question was simple and fair: what exactly would they be getting, in terms of skills, to prepare them for the workforce? I found myself uncertain about how to answer—because I, and many others in public health, have wrestled with this question for many decades [1, 2]. It has been a long-simmering problem that now has come to a boil, with national policymakers questioning whether a graduate public health degree should be treated as a professional degree worthy of resource support for students and other recognition alongside degrees such as those in medicine or law. With >35 000 students enrolled in accredited public health graduate programs worldwide each year, this is a serious concern for our public health workforce and the profession itself [3].

Public health leaders emphasize that MPH graduates are trained in food safety, clean water, and outbreak response [4]—exactly the kinds of tangible professional skills that matter. But, at the same time, these arguments lose force when the MPH is also described primarily as “a public health framing,” “the basics,” or “an understanding”—language that is unlikely to persuade students seeking concrete professional skills, or skeptical government officials. Those goals are valuable, but they are not sufficiently specific to function as proof of professional readiness. This can leave programs emphasizing basic knowledge about public health more than mastery of what public health professionals actually do and the skills required to do such work. Some undergraduate public health programs seem more applied and skills-forward than their graduate counterparts. That should give us pause. We are at risk of treating graduate public health education as if it were a continuation of undergraduate liberal arts education. And with full respect for the immense value of liberal arts learning—which I strongly support—that approach is a mistake for a graduate-level professional degree.

Graduate-level, professional public health education should not simply repeat what many students already encountered as undergraduates, whether in public health or related majors. We need to confront a longstanding concern: that public health, as a field, too often fails to clearly confer professional, job-ready skills at the graduate level. The cost of this gap is not abstract. It affects students’ workforce prospects, prospective applicants’ willingness to enroll, satisfaction of employers with our graduates, and our reputation and sustainability as a profession. Skills-based learning must become the bread and butter of MPH curricula [5], with an unapologetic focus on public health fieldwork, applied methods, and solutions-oriented practice.

Policymakers at the highest level have determined that an MPH does not satisfy the definition of a professional degree because, they argue, it is not required for entrance into a specific profession and does not lead to licensure or board certification [6]. Board certification verifies a person’s professional knowledge, skills, and abilities to perform a specific job or work in a specific field. It carries significant weight and recognition, assuring professional competence often alongside an academic degree. Public health realized decades ago that it needed to be recognized as a verifiable profession and instituted the National Board of Public Health Examiners Certification in Public Health [7].

Since its inception in 2008, >10 000 public health professionals have received National Board certification; however, <5% of MPH graduates have actually become certified. This low uptake suggests that the credential has not yet become a meaningful or expected standard within the public health profession. The problem is not necessarily with the certification itself, but rather with the absence of regulatory requirements comparable to those in fields such as medicine or nursing, in which licensure and board certification are more clearly tied to professional practice. In addition, most employers of MPH graduates have not consistently required or even strongly encouraged skills certification, further undermining its value and visibility. Until board certification becomes more obvious to graduate students and employers begin to demand it, the credential is likely to continue being viewed as an optional, $385 USD add-on rather than an essential marker of professional competency.

Ironically, the rise of artificial intelligence (AI) may be making the need for National Board certification more apparent. Employers are increasingly turning to their own in-house assessments of new MPH graduates before extending job offers, in order to determine whether candidates have truly mastered fundamental public health concepts and skills. Their concern is whether graduates can demonstrate genuine competency, or whether some may have relied too heavily on AI tools to complete their MPH coursework. Broader adoption of board certification could help address this problem by providing employers with a standardized measure of professional competence, sparing them the burden of developing and administering their own internal tests to ensure that they are hiring capable public health professionals. Although some public health leaders believe that our current MPH training alone is enough for our students, and indeed many have secured jobs in the past simply with an MPH [8], this may not be consistent with the goal of professional recognition by many outside the profession, including those in government and healthcare. Having a demanding, widely sought-after board certification could confirm important professional skills and distinguish the MPH from a host of competing graduate and professional degrees.

We need to ensure that MPH programs are not simply producing public health appreciators, but are also building a robust workforce of public health proceduralists: certified professionals whose skills are eagerly sought by employers and whose work has clear, measurable value for the health of the public. Although MPH graduates will likely work within teams in which expertise is shared, they should still be trained as individuals to collect data first-hand, analyse large publicly available datasets, respond to emerging health threats, implement health interventions, understand how major diseases and conditions operate, interact directly with people affected by those diseases and conditions, and use the most up-to-date tools in technology, epidemiology, statistics, graphics, and media [9]. That’s a lot. Unfortunately, MPH accreditation by the Council on Education for Public Health includes some, but few, of these on-the-ground skills as competencies and mostly emphasizes foundational knowledge [10].

Public health is already a professional degree, for many reasons [11]. A revival of the skills-first roots of public health will put the profession in a much stronger position to defend itself and show that it is prepared to protect and promote health, millions at a time, in countries around the world. Just as importantly, we will finally have a better answer for students asking the question that keeps coming back, year after year: what skills will I gain that allow me to actually make a positive change in the public’s health? If we can’t answer that, then we should be willing to ask the harder question of ourselves—otherwise, what’s the point?
